# Supplementary material for: Are carbon emissions trading and green financial instruments synergistic? -Comprehensive quantitative research based on content analysis
Source: PLoS One. 2024 Mar 7;19(3):e0298601. doi: 10.1371/journal.pone.0298601 (PMC10919601; doi:10.1371/journal.pone.0298601)
Supplement: S1 Data — (ZIP) [file pone.0298601.s001.zip › Data/地级市Z_F工作报告.zip!/╔╜╢1⁄2/╝├─■╩╨/└┤╫╘╣1⁄2╓┌║┼í╛┬φ┐╦╩2╛▌═°í┐_2018.docx]

各位代表：

现在，我代表济宁市人民政府向大会报告工作，请予审议，并请市政协委员提出意见。

一、2017年工作回顾

过去一年，全市上下以习近平新时代中国特色社会主义思想为指导，在省委、省政府及市委坚强领导下，按照“一二五六”目标思路，团结拼搏，真抓实干，经济社会发展取得新成绩。预计实现地区生产总值4620亿元、增长7%。一般公共预算收入385.7亿元、增长3%，扣除省市减税降费政策等因素增长13.9%。固定资产投资3474亿元、增长8.4%。规模以上工业利润、利税增长36%、31%，为10年来最高。存贷款余额首次突破5000亿元、3000亿元。新增境内外上市企业4家、挂牌企业75家，结束了6年无新增主板上市公司历史。常住人口城镇化率提高1.8个百分点、达到57.1%，增幅连续5年居全省前列。PM2.5浓度在京津冀“2+26”通道城市最低。为民所办十件实事全面完成。成功创建国家卫生城市，全国文明城市创建在2017年省级测评中获得第1名，城市形象、市民素质明显提升。

一年来，主要做了以下工作：

（一）加快新旧动能转换，新的经济增长点加速形成

供给侧改革成效明显。压减煤炭产能36万吨。房地产去库存1194万平方米，去化周期处于合理区间。综合为企业降成本77亿元，减免高新技术企业和小微企业税收70.1亿元，为历年来最高。实体经济新增贷款278.8亿元，同比多增123.3亿元。工业品销售率达到98.6%。规模以上工业企业资产负债率下降1.2个百分点。

传统产业提档升级。实施“千亿技改”“百企护航”等六大工程，完成技改投资1149亿元、增长22.9%，制造业利润、利税同比增长28.5%、23%。如意集团荣获中国质量奖提名奖。新增规上服务业企业275家，总数居全省第2位。新认证国家“三品一标”农产品61个，新增省级标准化生产基地19处。农作物综合机械化水平达到85%，获评全国“平安农机”示范市。济宁国家农科园通过验收，金乡入选首批国家现代农业产业园。

“四新经济”蓬勃发展。高新技术产业产值增长10.8%、提高6个百分点。信息产业加快发展，营收增长15%，高新区获批省三大大数据产业集聚区，华为云计算数据中心一期建成。电商交易额达到1600亿元、增长25%。编制完成新旧动能转换总体方案，实施首批“四新四化”项目364个，新开工亿元以上项目738个、同比增加410个，比亚迪产业基地开工建设，比克电池项目落户济宁，中建国际示范园进展顺利。落地开工PPP项目43个，是全省唯一获国务院表彰的市。

园区发展提速增效。出台加快园区发展实施意见，每年列支2亿元支持园区发展。新建园区道路210公里、基础配套工程173个、公共服务平台93个。园区增加值、固定资产投资增长10%、13%，高于全市3个、4.6个百分点。

（二）强化三大动力支撑，发展活力持续迸发

重点领域改革有序实施。深化行政执法体制改革，11个领域实现综合执法。128家市管企业完成公司制改制。完成规范化改制企业484家，是省定任务2倍。教育、卫生体制改革逐步深化，城市和县级公立医院全部建立法人治理结构。

“放管服”改革纵深推进。实施项目审批流程再造，推行“四个一”模式，削减行政权力事项50项，审批时限比法定时限缩短一半。推进“互联网+电子政务”，90%行政审批事项实现网上办理。健全“十张清单”制度，构建信用信息“一网三库一平台”，在全省率先形成贯通市县乡村的公共服务事项目录。

创新型城市加快建设。新增国家高新技术企业148家、省级以上科技创新平台30个，国家纺纱工程技术研究中心建成使用，全省首个环境类院士工作站落户济宁。新引进院士6人、入选省级以上人才工程44人，集聚海内外高层次人才2586人，高新区获批国家创新人才培养示范基地。

对外开放取得突破。实现进出口总额414.4亿元、增长15.2%，增幅6年最高。新设外商投资企业33家，实际利用外资增长17.9%。完成境外投资80.6亿元、居全省第1位。

（三）致力于城乡协调发展，重大工程重点项目提速推进

精致城市建设成效明显。实施重点城建项目179个、竣工107个，改造老旧小区121个、竣工107个，开工棚改7.2万户、建成6.9万户，超额完成省定任务。新开工地下综合管廊9.4公里，建成海绵城市19平方公里。新建公园绿地23处，升级改造主次干道106条、背街小巷203条，打通断头路25条。入选全国“城市双修”试点市、全国首批装配式建筑示范市、全国公共建筑能效提升重点市，城市规划工作是全省唯一受住建部表彰的市。

城乡环境面貌持续提升。完成美丽乡村建设总规编制，建成省级美丽乡村示范村45个、市级55个，在全省考核中位居前列。改造农村厕所49.4万户，开展城乡供水一体化试点，基本实现天然气“镇镇通”。曲阜市、金乡县成功创建国家园林城市，尼山镇入选全国特色小镇。

重大交通项目快速推进。跨湖高速、董梁高速、鲁南高铁等牵动性工程开工建设。机场迁建通过国家立项评估，基本具备开工条件。日兰高速济宁连接线、北二环东延竣工通车。泗河堤防主体工程全部完工。西二环、济宁大道西延加快实施。环湖大道东线微山段全面启动。胡集港等“七大港口”提速推进。城际公交实现“县县通”，镇村公交通达率、覆盖率分别达到100%和95%。

（四）实施五大攻坚，防风险筑底线取得新成效

生态环境持续改善。实施大气污染防治集中攻坚，整改突出环境问题1433个，完成燃煤锅炉超低排放改造176台、气代煤电代煤6万户，2069家“散乱污”企业、6006台燃煤小锅炉全部“清零”，秸秆禁烧工作受到省政府表彰。“蓝繁”天数同比增加27天，PM2.5、空气质量综合指数改善23.3%、11.5%，均居全省第1名；健全“治用保”水污染治理体系，开展面源污染专项治理，南四湖非法采砂全部清除，是中央环保督察转办信访案件最少的市之一，是中央环保督察组对山东反馈意见中唯一表扬的市。国家森林城市创建扎实推进，新造林20.9万亩，修复湿地10.6万亩，治理水土流失9.1万亩、采煤塌陷地2.3万亩。

安全生产态势平稳。严格落实部门、属地、企业责任，深入推进“素质固安”工程，持续强化“大快严”集中整治，狠抓化工产业安全生产转型升级，事故起数、死亡人数同比下降61.5%、34.2%，异地安监执法、安全责任边界厘清等经验做法全省推广。

金融风险防控有力。规范政府举债融资行为，开展互联网金融风险专项整治，严厉打击恶意逃废金融债务违法犯罪，“一企一策”制定担保圈应对预案，金融生态环境持续改善。

脱贫攻坚精准有效。“六大脱贫攻坚行动”统筹推进，投入扶贫资金2.9亿元，510个贫困村实现“五通十有”。实施产业扶贫项目208个，建设扶贫车间143个，梁山黄河滩区脱贫迁建工程全面启动，全市3.66万农村贫困人口实现脱贫，获省年度考核第1名。

社会大局和谐稳定。突出抓好十九大、“一带一路”峰会等重大活动安保维稳，实行战时指挥部机制，实现“六个不发生”目标，是全省六个先进市之一。深入开展矛盾纠纷大排查大化解专项行动，化解矛盾纠纷2560件。深化“天网工程”建设，加强公共安全监管，刑事、治安、火灾警情同比下降17.7%、22.4%、7.2%，平安济宁建设取得新成效。

（五）提高民生保障水平，群众幸福感获得感不断增强

就业创业量质齐升。全面落实就业创业扶持政策，就业补贴资金支出2.7亿元，新增城镇就业7.5万人、高技能人才9300人，就业创业培训7.7万人，2个创业孵化基地获评省级创业示范平台。

社保体系日趋完善。连续13年提高企业退休人员养老金标准，城乡低保实现“十二连增”。职工和居民大病保险最高报销额由45万元提高至65万元。新增养老床位5000张，获批全国居家和社区养老服务改革试点市、省医养结合示范先行区，跻身中国城市公益慈善百强榜18强、居全省第1位。

办学条件显著改善。大班额三年行动计划圆满完成，累计完成投资102.3亿元，新改扩建学校187所，竣工318.2万平方米，新增学位19.7万个，城镇小学、初中、高中大班额比例分别由2015年的51.6%、53.6%、61.4%下降到35.1%、40.4%和31.7%。14个县市区全部通过国家义务教育发展基本均衡县验收。职业教育和高等教育办学水平不断提升，曲阜师范大学新区启动建设，济宁医学院医药工程学院入驻高新区。

文化建设成果丰硕。省政府正式出台曲阜传统文化示范区建设规划。完成历史文化名城保护规划修编。曲阜市获评十大“中国年度文化影响力城市”。“三孔”彩绘、尼山圣境、鲁国故城遗址公园进展顺利，孔子博物馆、市文化中心主体完工。新改扩建村级综合文化服务中心3400处，实现二级文化馆县级全覆盖。文化产业增加值增长17.6%。

健康济宁扎实推进。市中医院新院试运营，市立医院开工建设。实施精准医疗“同质化”服务模式，建成医联体30个。70%妇幼健康服务机构、80%乡镇卫生院和社区卫生服务中心达到规范化标准。食品药品安全监管不断强化。国防动员、人民防空、民兵预备役、国家安全、外事侨务、统计、物价、粮食、地震、体育等工作进一步加强，广播电视、民族宗教、公积金、史志、档案、气象、老龄、残疾人和妇女儿童等事业取得新进步。

一年来，我们不断加强政府自身建设，严守党的政治纪律政治规矩，思想上政治上行动上同以习近平同志为核心的党中央保持高度一致。严格落实政府系统全面从严治党主体责任，扎实推进“两学一做”学习教育常态化制度化，干部作风不断转变。自觉接受人大法律监督和政协民主监督，办理议案建议170件、政协提案660件，办复率100%。自觉遵守中央八项规定精神，强化审计监督，坚决纠正“四风”，严惩腐败行为，树立了清正廉洁政府形象。

这些成绩的取得，是市委正确领导、科学决策的结果，离不开市人大、市政协的监督支持，凝聚着干部群众的心血汗水。在此，我谨代表市人民政府，向全市各族人民，向离退休老领导老同志，向驻济部队指战员、武警官兵、公安干警和人大代表、政协委员，向关心支持济宁改革发展的港澳台同胞、海外侨胞和国际友人，表示衷心感谢和崇高敬意！

各位代表，我们也清醒地看到，在发展道路上还面临不少困难和问题。主要是：新旧动能转换还不够快，发展质量效益还不够高。城乡建设还存在一些短板，承载能力有待增强。生态环境建设任重道远，安全生产、信访稳定、金融风险防控压力较大，脱贫攻坚成果还需持续巩固。县市区发展不平衡问题仍比较突出。民生事业与群众对美好生活的新期待还有不小差距。政府职能转变还不到位，作风建设还需要进一步加强。对此，我们一定高度重视，采取有力措施，切实加以解决。

二、2018年主要任务

今年是贯彻党的十九大精神的开局之年，是决胜全面建成小康社会的关键一年。做好今年政府工作，我们要把学习贯彻党的十九大精神作为首要政治任务，以习近平新时代中国特色社会主义思想为指导，加强党对经济工作的领导，坚持稳中求进工作总基调，统筹推进“五位一体”总体布局和协调推进“四个全面”战略布局，认真落实省第十一次党代会决策部署，按照市委“一二五六”目标思路，以供给侧结构性改革为主线，以新旧动能转换重大工程为统领，扎实做好稳增长、促改革、调结构、惠民生、防风险各项工作，努力实现高质量发展，为决胜“两个全面”战略部署奠定坚实基础。主要预期目标为：地区生产总值增长7%以上，一般公共预算收入增长6%左右，固定资产投资增长8%左右，社会消费品零售总额增长10%左右，城镇和农村居民人均可支配收入分别增长8%和8.5%左右。重点做好六个方面的工作。

（一）加快新旧动能转换，建设现代化经济体系

实施乡村振兴战略。坚持农业农村优先发展，编制完成乡村振兴战略五年规划。深化农业供给侧结构性改革，大力调整农业结构，因地制宜扩大特色产业规模，提高金乡大蒜、邹城食用菌、微山湖大闸蟹、鱼台大米小龙虾等优质品牌比重，新认定“三品一标”50个，打响“济宁礼飨”农产品公共品牌。开展粮食绿色高产高效创建，推进农作物生产全程机械化，加快畜牧业生态健康发展，粮食产量稳定在500万吨以上，确保全市粮食安全。大力发展农业“新六产”，培育休闲、创意农业，加快济宁、邹城国家农科园建设，新认定市级农业产业化龙头企业50家、农民示范合作社100家。提升美丽宜居乡村建设水平，推动全国特色小镇、重点示范镇加快发展，每个县市区重点培育1个示范镇，完成美丽乡村示范带和1500个村庄规划，建成美丽乡村达标村152个，覆盖率达到60%以上。实施济宁东部电网改造提升工程。启动农村清洁供暖三年行动计划，加快天然气“村村通”工程建设。推进城乡供水一体化发展，有效解决农村饮水安全。抓好“厕所革命”，全面完成农村无害化厕所改造任务。建设“四好”农村公路500公里，完善农村基础设施管理长效机制。加强农村公共文化建设，组织千场大戏进农村、万场演出惠民生、公益电影放映等文化惠民活动，村级综合文化服务中心基本实现全覆盖。大力开展移风易俗活动，让文明乡风吹遍乡村每个角落。培育农村人才队伍，探索建立高层次人才志愿服务基层制度，做好村和城市社区“两委”换届选举工作，深入推进农村“双创”活动，吸引在外务工人员、大学生回乡创业，培育新型职业农民2000人，造就一支懂农业、爱农村、爱农民的农村工作队伍。

聚力发展先进制造业。制造业是我市转型振兴的重要支撑，必须把推动制造业高质量发展作为强市之基，深入实施“中国制造2025”济宁行动计划，全力推进产业转型升级。主攻“四新四化”。实施新一轮千亿技改工程，市级层面筛选100个重大项目集中跟进，支持如意、华勤、太阳纸、东宏等优势企业向产业链价值链高端攀升。鼓励煤炭企业加快转型升级，推进安全绿色发展，支持兖矿清洁煤开发推广。启动“企业上云”行动，拓展物联网、大数据、云计算等现代信息技术在制造业全产业链集成运用，打造10条智能生产线，培育10个两化融合示范企业、10个智能装备产品、20个“上云”标杆企业，推动传统生产模式向“制造+服务”转型。做大做强六大千亿级产业。信息产业，深入实施“6501”三年行动计划，拓展与华为、中兴合作领域，启动5G通讯网络设施布局，加快济宁大数据中心和运河软件产业园建设，集中扶持高端光伏、LED、光通信、大数据、集成电路、智能终端等优势产业加快发展。高端装备制造产业，围绕工程机械、数控机床、精密制造、机器人、轨道交通等重点领域，突破关键技术、核心部件，健全协作配套体系，形成完备产业集群。支持高新区高效传动与智能铲运机械创新型产业集群加快发展。化工新材料产业，加快化工产业安全生产转型升级，推动化工产业向高端化、精细化、集聚化迈进；以凯赛、利特纳米、益大新材料等龙头企业为主导，建设绿色尼龙、石墨烯、针状焦等高端产业园。新能源汽车产业，依托比亚迪、比克电池、联诚等龙头企业，引进电机、电控等产业链项目，支持梁山专用车、重汽商用车加大新能源汽车研发生产，规划建设新能源汽车产业园，形成整车生产能力。高档纺织服装产业，以如意集团为引领，支持纺织服装企业向服务制造型发展，加快建设全国知名时尚纺织智造基地。新医药产业，以国家级生物技术产业基地为依托，扶持鲁抗、辰欣、希尔康泰等龙头企业加快发展，构建以高端制剂、中成药等为主导的产业体系，打造现代生物医药基地。强化园区主体。按照产城融合理念，厘清园区边界，提升规划水平，实现总规和专项规划全覆盖。完善基础设施及公共服务配套，加快园区智能化建设，重点打造2个示范智慧园区。设立50亿元产业发展引导基金，每个园区明确2-3个主导产业，形成主业突出、特色鲜明、协调配套的产业格局。加快园区体制机制创新步伐，激发园区发展生机与活力，努力把园区打造成“四新四化”的主战场，区域经济的增长极，产城融合的示范区。严格落实分类考核、分类推进政策，激励园区争先进位。支持兖州工业园、邹城开发区争创国家级开发区。鼓励大型企业围绕产业链兴建专业园区。打造强企方阵。启动“雁阵计划”，对年销售收入在50亿元以上“头雁”企业，建立市级领导“一对一”帮扶机制，鼓励企业全球配置资源，力争2家企业营收过500亿元、4家过100亿元。对销售收入过10亿元“隐形冠军”企业，集成要素资源重点支持，打造“雁阵”企业群体。加大中小微企业和科技型初创企业培育力度，重点破解融资、研发、人才等共性难题，对首次纳入规上企业统计范围的中小微企业给予支持奖励，逐步形成“头雁引领、雁阵跟进、群雁齐飞”格局。集聚资源要素。加大金融对实体经济支持，深化政银企对接，推进“个转企、小升规、规改股、股上市”，完成改制企业230家以上，新增“四上”企业1100家以上。鼓励有条件的企业发债融资，建立完善“投贷联动”机制，服务更多创新创业型企业。强化用地保障，让好的项目享受好的资源。严格耕地保护，严查违法占地，促进节约集约用地。实施“十百千”品牌培育工程，新增中国驰名商标、山东名牌产品10个以上。强化质量管理和知识产权保护，鼓励社会团体、行业组织、企业参与国家标准、行业标准修订。加强企业家队伍建设，组织科技型、成长型企业负责人赴境外培训，优选企业高管到高校研修、到世界500强企业总部学习锻炼，推行青年企业家“导师制”，做好接班企业家队伍培养，以一流企业家队伍打造一流企业。构建“敢亲”“真清”新型政商关系，保护企业家合法权益，为企业家干事创业营造良好环境。

提质发展现代服务业。大力发展生产性服务业。发挥“水陆铁空”交通运输优势，加快建设兖州北站物流园、现代粮食物流园、济矿梁山物流园等综合物流园区。推进传统商贸业转变经营模式，促进电子商务规范发展，支持亿矿网、蜗牛货车等本土电商做大做强，实现县域电商产业园全覆盖，交易额增长20%以上。加快发展研发设计、检验检测等科技服务业，推动服务业与制造业深度融合。繁荣发展生活性服务业。以创建国家全域旅游示范区为抓手，积极推进“旅游+”融合发展，突出抓好环微山湖生态旅游经济带规划建设，加快南阳古镇、济安台文化街区、济州古城等项目进度，整体提升乡村旅游发展水平，旅游消费总额增长10%以上。建立多主体供给、多渠道保障、租购并举住房制度，保持房地产市场平稳健康发展。融合发展医疗保健、养生康复等医疗服务业，建设鲁南生态康养基地。完善服务业发展政策。发挥任城区、邹城市省级服务业综合改革试点示范作用，落实服务业发展优惠政策，放宽市场准入，鼓励社会资本投向市政公用、家政服务、教育医疗等产业。培育服务业“四大载体”，抓好重点城区、园区、企业和项目建设，打造现代服务业发展新引擎。

（二）突出重点领域关键环节，持续推进改革创新扩大开放

加强供给侧结构性改革。以提高供给质量为主攻方向，大力破解无效供给，坚决淘汰落后产能和工艺，支持企业增品种、提品质、创品牌，提升核心竞争力。稳妥做好去产能工作，完成煤炭消费减量任务。因地制宜、分类施策加大去库存力度。全面落实支持国企改革、非公经济发展政策措施，有效降低企业人工成本、制度性交易成本、融资成本、用能成本。

深化重点领域改革。深入推进“放管服”改革，实施行政审批再提速、政务服务再优化等“六大工程”，力争80%以上审批事项实现“最多跑一次”。建立全市统一的政务大数据平台，部门主要政务信息资源实现共享。推进财税体制改革，调整完善市县财政体制，规范一般性转移支付管理。完成国家和省农村集体产权制度改革试点县任务，市级改革试点扩大到所有涉农乡镇。市属经营性国有资产全部纳入统一监管体系。支持高新区建设全面深化改革先行区。

提升创新发展能力。实行更加积极、更加开放、更加有效的人才政策，扎实推进国家创新型城市和省级人才改革试验区建设，支持园区建设“双创”基地。突出企业创新主体地位，引导企业联合大院大所大学共建产学研平台。实施鲁西科学发展高地人才支持计划和“创新行动·智绘济宁”三年行动计划，完善顶尖人才引进“一事一议”政策，新引进创新创业领军人才20名、高技能人才5000名、院士2名以上。强化科技型企业扶持，加大减税降费力度，建立投融资支持体系和风险分担机制，营造一流创新创业环境。

构建开放新格局。坚持世界眼光、国际标准，以海纳百川的胸襟、敢为人先的气魄，以改革开放40周年为新起点，全力推进新一轮高水平对外开放。深度融入“一带一路”建设，支持煤炭、建材、化工、纺织等优势产能“走出去”，以增强核心竞争力为目标，开展境外并购重组，发展服务外包、信息技术、文化旅游等服务贸易。积极争取设立兖州直通“中欧班列”站点。坚持把招商引资作为发展的生命线，按照“招大引强选优”思路，注重招商引资与招才引智相结合，围绕建链补链强链，大力开展产业招商、以商招商、资本招商，探索“房东+股东”模式，规划建设一批标准化厂房，培育一批专业化招商人才，密切与世界500强、中国500强企业对接，办好珠三角、长三角和欧美、日韩、香港等境内外招商活动，集中突破龙头企业引进，着力抓好产业项目落地，力争到位市外国内资金400亿元以上，利用外资增长10个百分点以上。

（三）加快推进新型城镇化，建设精致宜居幸福城市

优化城镇体系结构。坚持中心城区突破、都市区融合、城乡一体发展思路。在中心城区布局上，统筹生产、生活、生态“三生”空间，优化产业布局，完善服务功能，加快环城生态绿带、环城路网规划建设，做大做强中心城区。在都市区融合上，按照生态、产业、设施、组织“四个一体化”发展路径，不遗余力推进“济兖邹曲嘉”加快融合。以大运河为纽带，推进太白湖新区、经开区、任城区、嘉祥县联动发展；以泗河为廊道，加速兖州区、高新区和邹西大工业板块紧密对接。在城乡一体发展上，突出城市片区、专业新城、特色小镇、美丽乡村4个重点，抓好项目规划建设，推动老城区、新城区、功能区和城乡协调发展。

扎实推进城市“双修”。实施城区绿化三年行动计划，今年新增绿化面积278万平方米，完成凤凰台公园、儿童公园主体建设。加快环城塌陷地治理，实施“五河”提升工程，逐步形成“一环八水绕济宁，十二明珠映古城”水城风貌。提升教育、医疗、文化等公共服务水平，加快打造15分钟生活圈。依托棚改做好老城区功能疏解“减法”和公共空间“加法”文章，改造老旧小区64个，开工棚改项目97个、5.8万套。推进“大交通”“微循环”和慢行系统规划建设，改造主次干道13条，打通断头路19条；开工建设王母阁路跨线桥，车站南路、火炬路跨线桥完成主体工程。完善市政基础设施，新增城区天然气用户1万户。建成海绵城市9平方公里、综合管廊8公里、雨污分流30公里。

完善市域大交通体系。开工建设济宁新机场、机场高速、鲁南高铁西段，完成跨湖高速、董梁高速、环湖大道东线路基桥涵，推进都市区“一环六联”快速路网建设，力争新外环建成通车。改造提升京杭运河主航道、湖西航道。

构建全域大生态系统。统筹山水林田湖草生态建设，完成山体修复3.2万亩，治理采煤塌陷地3.8万亩，保护修复湿地10万亩。泗河闸坝全面开工建设。启动引汶补源入济和引黄工程。全面攻坚城区、城郊、山区、镇村等重点区域绿化，新造林15万亩以上。扎实做好国家森林城市迎查验收工作。实施南四湖自然保护区、南水北调东线生态带、黄河生态带水系绿化，高标准建设生态廊道。

提高精细化管理水平。严格落实导则，健全“标准化、规范化、数字化”城市管理长效机制。推行道路保洁“以克论净”模式，加快数字城管智慧化升级，增强城市服务保障能力。完善城市智能交通体系，提升疏堵保畅水平。强力推进违法建设拆除、餐饮油烟治理、渣土管理等攻坚行动，全面实施餐厨垃圾集中收运处理。下功夫规范提升物业管理水平，努力实现社区物业管理全覆盖。大力整治脏乱差现象，打造洸河路等10条示范街。巩固国家卫生城市创建成果，矢志不渝推进全国文明城市创建。

（四）发挥文化资源优势，加快建设首善之区文化强市

打造儒家文化传播高地。依托孔子研究院、孟子研究院等儒学机构，引进一批儒学大家，培养一批青年人才，形成一批权威成果。办好国际孔子文化节、尼山世界文明论坛等重大活动，提升儒学研究推广话语权。争取知名高校在我市设立国学分校。加强干部政德教育基地建设，提升办学质量规模，增强对外影响力。

打造文化产业发展高地。积极推进曲阜传统文化示范区规划建设，尼山圣境一期建成开放。挖掘运河文化内涵，争创全国首批运河文化建设示范市。大力发展文化创意、数字传媒等新兴产业，推进爱奇艺“双创”基地建设，搭建文创产业“五大平台”，建设创意文化产业园，力争文化产业增加值突破200亿元。

打造精神文明道德高地。常态化开展儒学六进、道德提升等七大工程，推动优秀传统文化深入人心。广泛开展精神文明创建，培育150个示范单位、200个示范村居、1.2万个示范户，评选一批道德模范、济宁好人。加强文艺人才队伍建设，培育文化名人，创作文艺精品。建成市文化中心。抓好第二轮修志工作。

（五）聚焦守底线防风险，保持全市大局和谐稳定

筑牢安全生产防线。坚持源头防范和系统治理，持续开展“大快严”专项整治，严格落实“三个必须”，持之以恒实施“素质固安”工程，大力提高防灾救灾能力，确保全市安全生产形势持续稳定。

提升生态环境质量。牢固树立“绿水青山就是金山银山”绿色发展理念，坚决抓好中央环保督察整改工作。打赢蓝天保卫战，持续开展集中攻坚，力争PM2.5、PM10浓度和重污染天数分别再降10%，让“济宁蓝”成为城市新名片。深入实施“水十条”，严格落实“河长制”“湖长制”，打造“治用保”污水治理升级版。全面落实“土十条”，开展重污染土壤治理和修复试点，搞好固体废物管理和综合利用。严控“双高”行业，万元GDP能耗下降3.66个百分点以上，完成第二次污染源普查，推动形成绿色发展方式和生活方式，努力使济宁的天更蓝、地更绿、水更清、空气更清新。

巩固脱贫攻坚成果。建立专项扶贫、行业扶贫和社会扶贫大格局，关注老弱病残等特殊贫困群体，聚焦湖区、滩区、山区等重点区域，注重扶贫与扶志、扶智相结合，激发贫困人口内生动力。加大财政投入，拓宽增收渠道，建立长效机制，保证现行标准下的脱贫质量，做到脱真贫、真脱贫、防返贫。

强化金融风险防控。加强薄弱环节监管，稳妥推进企业风险化解，严厉打击逃废金融债务、非法集资等违法违规金融活动，加大互联网金融风险整治，大力压降不良贷款，维护良好金融生态环境。强化政府债务限额管理和全口径监测，妥善化解存量，严格控制增量，建立长效机制，确保债务率保持合理水平。

加强社会治理创新。做好社会治安、信访维稳、网络舆情等工作，确保社会大局和谐稳定。坚持法德兼治，抓好“七五”普法，全面提升尊法学法守法用法意识和能力。深化军民融合发展，争创全国双拥模范城“八连冠”。提升民族宗教、外事侨务和防震减灾等各项工作水平。

（六）顺应人民对美好生活向往，提高保障改善民生水平

推动更高质量就业。实施更加积极就业政策，推进创新创业，新增城镇就业7万人，扶持创业1.4万人。建立全方位公共就业服务体系，健全职业培训、创业扶持、就业援助机制，强化对城镇失业人员、高校毕业生、农民工等重点群体就业帮扶。实施劳动技能提升工程，职业技能培训6万人。

完善社会保障体系。实施全民参保计划，强化社保扩面征缴，落实基本养老保险待遇合理调整机制，实行居民基本医疗保险市级统筹，推行职工长期护理保险。积极推进全国居家和社区养老服务改革试点市、省医养结合示范先行区建设，新建城市社区日间照料中心30个，村级互助养老院100个，新增养老服务床位5000张，提升养老服务质量。

办好人民满意教育。建立消除大班额长效机制，完成“全面改薄”收尾任务，新改扩建中小学校60所，新增校舍面积60万平方米、学位3万个，努力解决中小学生课外负担重、“择校热”等突出问题。扩增高中阶段教育资源，优化中职和普通高中比例。大力发展学前教育，抓好普惠性幼儿园建设，新改扩建幼儿园60所。实施特殊教育二期提升计划，完善困难学生资助体系。加强教师队伍培训管理，弘扬优秀师德师风，提高教育教学质量。推进名校带动战略，加大优质教育资源引进力度，支持驻济高校建设，完善职业教育和培训体系，提升高等教育办学水平。

推进健康济宁建设。加快市立医院建设，完成市中医院新院配套工程。健全分级诊疗和药品供应保障制度，全面实施药品“两票制”。启动济宁卫生学校新校区建设。适应两孩生育高峰，增加产科、儿科医护人员和床位设施。推进基层医疗卫生机构标准化创建，90%以上乡镇卫生院和社区卫生服务中心达到标准，家庭医生签约服务覆盖率达到40%以上。深化计划生育管理服务改革，出生人口性别比稳中有降。强化“食安济宁”建设，全域推进“双安双创”工作。深入开展全民健身运动，营造健康生活方式。

三、全面加强政府自身建设

新时代要有新气象新作为。做好政府工作，必须持续强化自身建设，提高政治站位，增强抓改革促发展能力，建设担当实干、作风清廉干部队伍，奋力开创政府工作新局面。

旗帜鲜明讲政治。深入学习贯彻党的十九大精神，增强“四个意识”，强化“四个看齐”，更加自觉以习近平新时代中国特色社会主义思想武装头脑、指导实践、推动工作，更加自觉同以习近平同志为核心的党中央保持高度一致。扎实推进“两学一做”学习教育常态化制度化，开展“不忘初心、牢记使命”主题教育，不断提高政治觉悟和政治能力。坚持党对一切工作的领导，坚决贯彻市委决策部署，确保各项工作落实、落细、落地。

依法行政履职责。坚持依宪施政、依法行政，把政府工作全面纳入法治轨道。严格执行市人大及其常委会决议决定，自觉接受市政协民主监督，提高议案建议和政协提案办理质量。健全重大行政决策程序，加强调查研究，强化统计监测，实现政府决策科学化、社会治理精准化。深化行政执法和监督体系改革，落实执法责任制和责任追究制，让权力在阳光下运行。

勇于担当重实干。坚持“干”字当头、“实”字托底，不驰于空想、不骛于虚声，一步一个脚印，踏踏实实干好工作，切实做到“说了算、定了干、按期完”。完善容错纠错机制，为担当者担当，为干事者撑腰。自觉加强政治、业务知识学习，提升干部队伍专业化素养，不断增强改革发展能力。下决心精简会议文件，集中全部精力抓落实、促发展。严格绩效管理，加大监督检查，全面整治庸政懒政怠政，努力打造真心为民、务实高效的服务型机关。

从严治政筑底线。自觉遵守《准则》《条例》，严格落实中央八项规定实施细则精神，驰而不息纠正“四风”，“三公”经费零增长。健全廉政风险防控机制，聚焦公共权力、公共资源、公共资金、扶贫攻坚、工程招投标等重点领域，严查不正之风和腐败问题，营造风清气正的政治生态。

各位代表！实干成就梦想，奋斗赢得未来。让我们更加紧密地团结在以习近平同志为核心的党中央周围，在中共济宁市委坚强领导下，撸起袖子加油干，不断推动济宁更高质量、更有效率、更加公平、更可持续发展，为实现“两个全面”任务目标、谱写新时代济宁社会主义现代化建设新篇章而努力奋斗！
